# Plastic additives: challenges in ecotox hazard assessment

**DOI:** 10.7717/peerj.11300

**Published:** 2021-04-16

**Authors:** Andrew Barrick, Olivier Champeau, Amélie Chatel, Nicolas Manier, Grant Northcott, Louis A. Tremblay

**Affiliations:** 1 Cawthron Institute, Nelson, New Zealand; 2 Université Catholique de l’ Ouest, Angers, France; 3 French National Institute for Industrial Environment and Risks, Verneuil en Halatte, France; 4 Northcott Research Consultants, Hamilton, New Zealand; 5 University of Auckland, Auckland, New Zealand

**Keywords:** Emerging contaminants, micro(nano)plastics, Additives, Multiple stressors, Plastic fragmentation, Chemical functions

## Abstract

The risk of plastic debris, and specifically micro(nano)plastic particles, to ecosystems remains to be fully characterized. One particular issue that warrants further characterization is the hazards associated with chemical additives within micro(nano)plastic as they are not chemically bound within the polymers and can be persistent and biologically active. Most plastics contain additives and are therefore potential vectors for the introduction of these chemicals into the environment as they leach from plastic, a process that can be accelerated through degradation and weathering processes. There are knowledge gaps on the ecotoxicological effects of plastic additives and how they are released from parent plastic materials as they progressively fragment from the meso to micro and nano scale. This review summarizes the current state of knowledge of the ecotoxicity of plastic additives and identifies research needs to characterize the hazard they present to exposed biota. The potential ecological risk of chemical additives is of international concern so key differences in governance between the European Union and New Zealand to appropriately characterize their risk are highlighted.

## Introduction

Plastics were revolutionary inventions that fundamentally defined the 20^th^ and 21^st^ centuries by providing inexpensive, lightweight materials with nearly inexhaustible applications. Plastics provided significant societal and economic benefits to humanity which dramatically improved quality of life (Andrady & Neal, 2009; Thompson et al., 2009). There are international policies to mitigate release but plastic wastes are often mismanaged and end up in the environment posing a risk to ecosystems. This includes the increasing prevalence of micro(nano)plastics (MNPs) which has led to intensive research in recent decades (Oliveira & Almeida, 2019; Ritchie & Roser, 2020).

A general consensus is that any particle <5 mm in size is considered a microplastic and particles <1 µm are considered a nanoplastic but there are disagreements in the literature about the specific size distinction (Arthur, Baker & Bamford, 2009; Baun et al., 2009; Phuong et al., 2016; Frias & Nash, 2019; Hartmann et al., 2019; Fred-Ahmadu et al., 2020). Plastics in the micro scale, and to a lesser extent nano scale, can enter the environment as intentionally manufactured products (termed primary and including pre-production knurdles and microbeads from synthetic textiles), or as larger items that breakdown and fragment via physical and chemical processes (termed secondary, typically derived from meso-plastic pollution) (Cole et al., 2011; Hermabessiere et al., 2017). In recent years, numerous ecotoxicity studies have been conducted to assess whether MNPs pose significant environmental harm, often with conflicting results (Beiras et al., 2018; Franzellitti et al., 2019; Revel et al., 2020a). Questions have been raised as to whether the environmental risk of microplastics is being over estimated relative to other anthropogenic stressors (Burton, 2017). One of the main questions is whether MNPs inherently pose a significant environmental risk or if there are other facets, such as chemical accumulation of contaminants to surface of the particles or the presence of chemical additives, modulating their ecotoxicity.

It has been proposed that MNPs can have a trojan horse effect by accumulating chemicals present in the environment through adsorption and absorption processes, providing a novel vector for biological uptake (Kwon et al., 2017; Fred-Ahmadu et al., 2020). This can lead to the concentration of ambient contaminants and potentially increase the load to biota that ingest plastic particles. The risk of this mechanism remains to be fully characterized as many earlier studies focused on MNPs rather than focusing on associated contaminants (Burton, 2017). While hypothetically possible, it is difficult to ascertain how often this effect naturally occurs in the environment but given the exponential increase in plastic use, and the projected concomitant increase in the quantity MNPs in the environment, this remains an emerging concern that warrants investigation.

Another potential and, until recently, overlooked cause of micro(nano)microplastic ecotoxicity are the additives intentionally added to plastic polymers. Plastic polymers are rarely used in their pristine form and incorporate chemical additives to give specific functions to the material (Hahladakis et al., 2018; Fred-Ahmadu et al., 2020). Many chemical additives are categorized on the basis of key functional properties they impart in the finished material or article, e.g., improved processability, surface protectors/modifiers, material protectants, physicochemical property augmenters and functionalizing agents (Hansen, Nilsson & Slot Ravnholt Vium, 2014; OECD, 2019). Additives are rarely chemically bound to plastic polymers and have the potential to leach from plastic as the material degrades and weathers. The ecotoxicity of some chemical additives has been characterized, especially those classified as persistent, bioaccumulative or toxic (PBT) or as persistent organic pollutants (POPs), but the subsequent ecological risk they present as a component of plastics remains to be ascertained (Bejgarn et al., 2015; Li et al., 2016; Vázquez-Morillas et al., 2016; Durán & Beiras, 2017; Kim et al., 2019; Capolupo et al., 2020; Schiavo et al., 2020). One of the challenges of assessing the risk of chemical additives in plastic pollution is the ability to discriminate between their impacts and those of ambient contaminants that have subsequently been accumulated by the plastic. This conundrum is a major contributor to the need to assess whether or not ecological hazards of plastic pollution requires a need for novel approaches when assessing their potential risk (Fred-Ahmadu et al., 2020). Importantly, the link between polymer types or the same polymer composed as different products, and the release of additives needs to be investigated as different polymers and/or final products may require different types and amounts of additives to achieve comparable functionality (OECD, 2019).

A few review papers on MNPs have highlighted the most common groups of additives in plastics and the need to investigate their ecotoxicological hazards as there is currently limited data available (Hermabessiere et al., 2017; Hahladakis et al., 2018; Franzellitti et al., 2019; Fred-Ahmadu et al., 2020). As with manufactured nanomaterials, there are additional technical considerations to address when assessing or comparing the environmental risk of different plastic polymers. For example, differential buoyancy will influence mobility and transport, thereby influencing their respective fate and accumulation in different environmental compartments with particles ultimately ending up in the sediment of receiving environments (Erni-Cassola et al., 2019).

The aims of this review were to summarize current knowledge on the ecotoxicity of plastic additives and identify knowledge gaps and future research needs to assess the risks these chemicals in MNPs pose to exposed ecosystems and biota. We also considered differences in research needs between the European Commission, one of the most proactive regions investigating environmental risk, and New Zealand, a country with a reputation of strong environmental protection.

## Survey methodology

References for the present manuscript were identified through the assistance of google scholar as well ResearchGate to identify publications pertinent to the subject material. Pertinent articles were obtained from various literature databases including Science Direct, PubMed, Wiley, Springer, ACS and Taylor&Francis journals as well government databased such as New Zealand’s Environmental Protection Authority, Environment Canada, INERIS substance fact sheets, ECHA’s registered chemical fact sheets, US EPA ecotox knowledgebase and pesticideinfo.org. Data on select plastic additives was collated by conducting a review of publicly available regulatory and commercial databases. Search criteria included the following terminology: targeted searches for each specific chemical using the following key words separately: chemical name, CAS number, plastic additive toxicity, microplastic additive ecotoxicity and microplastic additives. Articles that related to additive toxicity towards terrestrial, freshwater and marine organisms were used in the analysis whereas articles that pertained specifically to environmental release of additives were not included used in the analysis.

### Overview of ecotoxicity on micro(nano)plastics and current limitations

In the past ten years, there has been a growing interest in assessing the potential environmental implications and ecotoxicity of MNPs as highlighted by a number of reviews (Galloway & Lewis, 2016; Lambert, Scherer & Wagner, 2017; Burns & Boxall, 2018; Franzellitti et al., 2019). Much of this research conducted is underpinned by lessons learned with engineered nanomaterials on the effects of size, shape and composition and their relationship to colloidal stability (Rist & Hartmann, 2018). While the initial principles are applicable, there are additional considerations of plastics that prevent direct comparisons. Plastic polymers are often not comparable to one another as there are key differences in physicochemical properties, such as density and porosity, between polymer types, making it difficult to establish clear reference materials for hazard assessment.

Many studies have used pristine plastic particles obtained from commercial products in their experiments rather than particles derived from fragmentation typically encountered in plastic pollution, thereby limiting the environmental relevance of experimental outcomes (Sussarellu et al., 2016; Magni et al., 2018; De Felice et al., 2019; O’Donovan et al., 2019). There has been significant progress made in understanding the toxicity of plastic particles themselves, including a growing body of research investigating environmentally representative plastics, often with mixed results (which may be attributed to the large variation in MNPs and measured endpoints) regarding effects in both terrestrial and aquatic environmental compartments (Connors, Dyer & Belanger, 2017; Booth et al., 2019; Revel et al., 2020a, 2020b). Research on more environmentally representative, irregular pieces of plastics composed of polyethylene terephthalate (PET), polyvinyl chloride (PVC), polyethylene (high density and low density) and some polyamides is also available, but the use of the outcomes is often limited due to inconsistent results and an absence of standardized methods.

**Figure 1:**
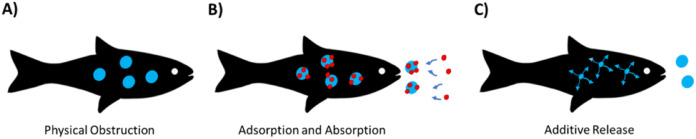
Primary methods of micro(nano)plasticecotoxicity. (A) Obstruction due to physical uptake of plastic particles, (B) adsorption and absorption of chemicals in the environment and (C) release of chemical additives.

When considering the potential ecotoxicity of MNPs, there are three main aspects of toxicity that are currently considered: (i) effects of plastic particles (mechanical and displacement of nutrients), (ii) absorption/adsorption of ambient organic and metal contaminants from the environment and (iii) chemical additives incorporated into plastic during production that can potentially be released after ingestion (Fig. 1).

### Physical and effects of micro(nano)plastics in the environment

The prevalence of MNPs has been extensively investigated and widely reported but conclusions on whether or not they are harmful to the environment remains to be confirmed (Ma et al., 2020). In general, work on MNPs has demonstrated that a range of adverse effects can occur from exposure to plastic particles ranging from populational effects (decrease in fertility and decline in foraging behavior) to transcriptional responses linked with immune and detoxification pathways (Avio, Gorbi & Regoli, 2017; Anbumani & Kakkar, 2018). Research on aquatic organisms demonstrated that both micro and nano plastics produce effects at sub individual levels of biological organization with limited impact due to polymer type, suggesting that many observed effects may be due to processes of physical obstruction. These include blockages and internal abrasion of tissues, like digestive systems, can be linked to reduced energy intake and inflammation (Besseling et al., 2013; Cole et al., 2013; Wright, Thompson & Galloway, 2013; Guzzetti et al., 2018; Trevisan et al., 2019; Yuan et al., 2019; Revel et al., 2020b). Similarly, assessments made with terrestrial organisms have demonstrated that plastics particles can interact with certain species via ingestion (worms and gastropods) leading to mechanical alterations, such as gut obstruction, which can decrease food assimilation and result in growth inhibition (Huerta Lwanga et al., 2016; Rodriguez-Seijo et al., 2017; Zhu et al., 2018; Wang et al., 2019; Jiang et al., 2020). Presently, linkages to physical obstruction are implied and work needs to be conducted to demonstrate this relationship. It has however been identified that size, type of polymer used, density/crystallinity, morphology (fragments, film, spheres, foams…), surface chemistry and morphology all influence ecotoxicity (Lambert, Scherer & Wagner, 2017).

### Adsorption and absorption of environmental contaminants to micro(nano)plastics

There are concerns over the potential role of MNPs in accumulating ambient organic and metal contaminants and providing a vector for their delivery to biological tissue, leading to a “Trojan-horse” effect (Regoli et al., 2017). The hypothesis that MNPs are novel vectors for anthropogenic contaminants that increases the uptake by aquatic organisms and ultimately humans is a topic that has been both supported and challenged in research and review papers (Smith et al., 2018; Akoueson et al., 2020; Walkinshaw et al., 2020). In the aquatic environment, there is evidence that plastics can interact, through adsorption and absorption, with numerous classes of anthropogenic contaminants, including present in the water column (Holmes, Turner & Thompson, 2012; Rochman et al., 2013; Brennecke et al., 2016). Organisms can be exposed to contaminants at concentrations that they would otherwise not interact with and may not be able to effectively mitigate and cope with stress. This effect has been previously demonstrated under laboratory conditions but the applied experimental parameters (concentration of chemical, mass and/or number of micro(nano)particles, contact times) have been atypical of conditions of exposure in receiving environments and further investigations are required before this hypothesis can be proven or disproved (Batel et al., 2016; Syberg et al., 2017; Li, Zhang & Zhang, 2018).

In addition to potentially increasing the exposure of biota to anthropogenic contaminants, plastic particles can alter the distribution of contaminants within aquatic organisms. For example, the ingestion of a mixture of polyethylene microplastic beads and silver (Ag) in fish leading to a significant reduction of Ag uptake but an increase in the proportion of Ag accumulating in the intestines (Khan et al., 2015; Luís et al., 2015; Ferreira et al., 2016; Fonte, Ferreira & Guilhermino, 2016). The potential bioavailability of chemicals accumulated from the environment onto MNPs and accompanying exposure to biota also requires consideration (Moore, 2008; Cole et al., 2011; Regoli et al., 2017; Bour et al., 2018; JPI Oceans, 2018).

### Plastic additives

Assessment of MNP ecotoxicity is not only challenged by the influence of polymer type, size and shape, but also by the sheer number, composition, and concentrations of chemical additives they contain. A large number of chemical additives incorporated into plastic are industrial chemicals produced in significant quantities such that they meet the definition of high production volume chemicals (Nikfar et al., 2014). Many find broad utility within a wide range of industrial applications. It is therefore perplexing there is limited information globally regarding their entry into the environment.

In a chemical sense the concentration of additive chemicals is very high in plastic (percent of weight) while the risk is often considered to be low due to the assumption there is negligible release post manufacture. The concentration of additives in plastic is considerably greater (orders of magnitude) than that of anthropogenic chemicals that are ‘sorbed’ to plastics in the environment (Hermabessiere et al., 2017; Hahladakis et al., 2018). Therefore the chemical additives in plastic could present a considerably higher potential risk to organisms exposed to them in the environment (Murphy, 2017). Given the amount of plastic entering the environment and their demonstrated persistence, associated additives could potentially be an underrepresented and poorly understood risk.

Additives are defined as any substance intentionally added to plastics to achieve a physical or chemical effect during the processing of a material or to impart functional properties to meet the requirements of the final products (ECHA, 2019a). The physicochemical properties of MNP particles can be altered by the additives they contain which in turn can alter the ways in which they behave in, and interact with, the environment (Rist & Hartmann, 2018). There are several reviews including the European Chemical Agency’s (ECHA) mapping exercise on plastic additives, that have provided robust descriptions on the diversity of additives as well as their main functions in plastic polymers (Hansen, Nilsson & Slot Ravnholt Vium, 2014; Hermabessiere et al., 2017; Hahladakis et al., 2018; ECHA, 2019b; OECD, 2019a). While chemically diverse, additives can be grouped based on key functional properties with concentrations in polymers often varying based on property (Table 1).

**Table 1:** Common functions of plastic additives.

Additive classifications	Sub classification	Typical concentrations (% w/w)	Description
Additives for processing	Plasticizer	10–70	Improves the fluidity of plastics during processing and flexibility at room temperature. Used extensively in polyvinyl chloride (PVC) molding
	Lubricants	0.1–3	Prevents the adhesion of plastics to the surface of metal molds and to each other, improve the fluidity of plastics, and reduce friction during melting and molding plastics
	Blowing (foaming) agents	Dependent on density of foam	Foam molding
Surface protectors/modifiers	Antistatic agents	0.1–1	Prevent static electrification of electrical insulators. Classified into coating agents and blending agents. Surfactants are often used.
	Antifriction agents	0.1–2	Antifriction agents reduce the surface friction coefficient.
	Adhesion improving agents	0.1–2	Adhesion-improving agents improve the adhesiveness of the surface of plastics
	Anti-fog additives	0.1–2	Hydrophobic surfaces permit condensation, leading to loss of translucency. Surfactants prevent fogging
Material protectants	Antioxidants	0.05–3	Some plastics produce radicals in response to heat and/or light. Antioxidants prevent oxidation and deterioration caused by heat during processing.
	Light stabilizers	0.05–3	Light stabilizers prevent oxidation caused by light during the service life of a plastic product.
	Ultraviolet absorbing agents	0.05–3	UV-absorbing agents prevent the breakage of molecular bonds by UV light and the generation of radicals
	Thermostabilizers	0.05–3	Thermostabilizers inhibit discoloration caused by the HCl produced from vinyl chloride resin because of heat during processing
	Biocides	0.001–1	Prevents degradation of plastics from microorganisms, often used with other additives
Physicochemical property augmenters	Flame retardants	12–18	Added to combustible plastics
	Fillers	0–50%	Various fibers and powders improve the strength of plastics
	reinforcement materials	15–30%	
Colorants	Soluble	0.25–5	Add color and make plastics light resistant.
	Organic pigments	0.001–2.5	
	Inorganic pigments	0.01–10	

**Note:**

Classifications for commonly used additives in plastic polymers. Information is adapted from Hansen et al. (2013), OECD (2019). Polyethylene—Low Density Polyethylene (LDPE) and linear low density (LLDPE), Polyethylene: HDPE (High Density), Polypropylene (PP), Polystyrene (PS), Expanded Polystyrene (EPS), Acrylonitrile butadiene styrene (ABS), Polyvinylchloride (PVC).

Many additives are not chemically bound to the polymers of plastics and can leach out, potentially providing a source of high production volume industrial chemicals into the environment (Hansen, Nilsson & Slot Ravnholt Vium, 2014). Some of the more commonly used additives (brominated flame retardants, phthalates, nonylphenols, bisphenol A and antioxidants) have been identified as POPs and are already subjected to restricted use in some countries as a result of the demonstrated PBT and/or endocrine disrupting properties (Hansen, Nilsson & Slot Ravnholt Vium, 2014; Hermabessiere et al., 2017; OECD, 2018). Many studies assessing the leaching of plastic additives have focused on chemicals like bisphenol A and phthalates due to the existence of extensive historical data sets and demonstrated modes of toxicity (Oehlmann et al., 2009; Kim et al., 2019).

Additive chemicals can also undergo transformation in receiving environments and degrade into metabolites with varying mechanisms of toxicity that are currently poorly understood. The ecotoxicity of several common plastic chemical additives in MNPs leachate has been characterized with results suggesting that additives can lead to neurotoxicity, inflammation, alteration to lipid metabolism and can have carcinogenic effects (Capolupo et al., 2020; Jeong & Choi, 2020). There is limited ecotoxicity data for many additives in part to their low production volumes and limited information on their fate in the environment. Several recent projects through the Joint Programming Initiative Healthy and Productive Seas and Oceans (JPI Oceans) have investigated the hazards of additives and identified that many studies focused on the hazards of pristine plastics with limited focus on the risk of their additives (JPI Oceans, 2020).

There are various recent initiatives around the world to better characterize plastic additives including the mapping exercise by ECHA (ECHA, 2019b). This initiative is focusing on compiling information on plastic additives produced in high volumes for commercial use and identify the degree to which they might be released from the plastic polymers across their life cycle. Additives on the list are classified based on key functions they impart to plastics as well as information regarding the concentrations used. The aim of this initiative is to establish a database to identify additives that are of potential environmental concern and select priority candidates for further investigations. Of the number of additives identified, up to 65% were currently under regulatory review by ECHA for use in the European Common Market. It is important to note that the list is selective and limited in scope as numerous plastic additives were not considered, mainly due to lack of information. One of the challenges identified in the exercise was that many industrial manufacturers did not provide comprehensive records on chemical additives, including concentrations, incorporated into plastic polymers (ECHA, 2019a). The absence of this data limits the ability to characterize the potential leaching of additives from various plastic polymers and estimate the corresponding load that could be released into the environment from manufactured plastic products.

**Figure 2:**
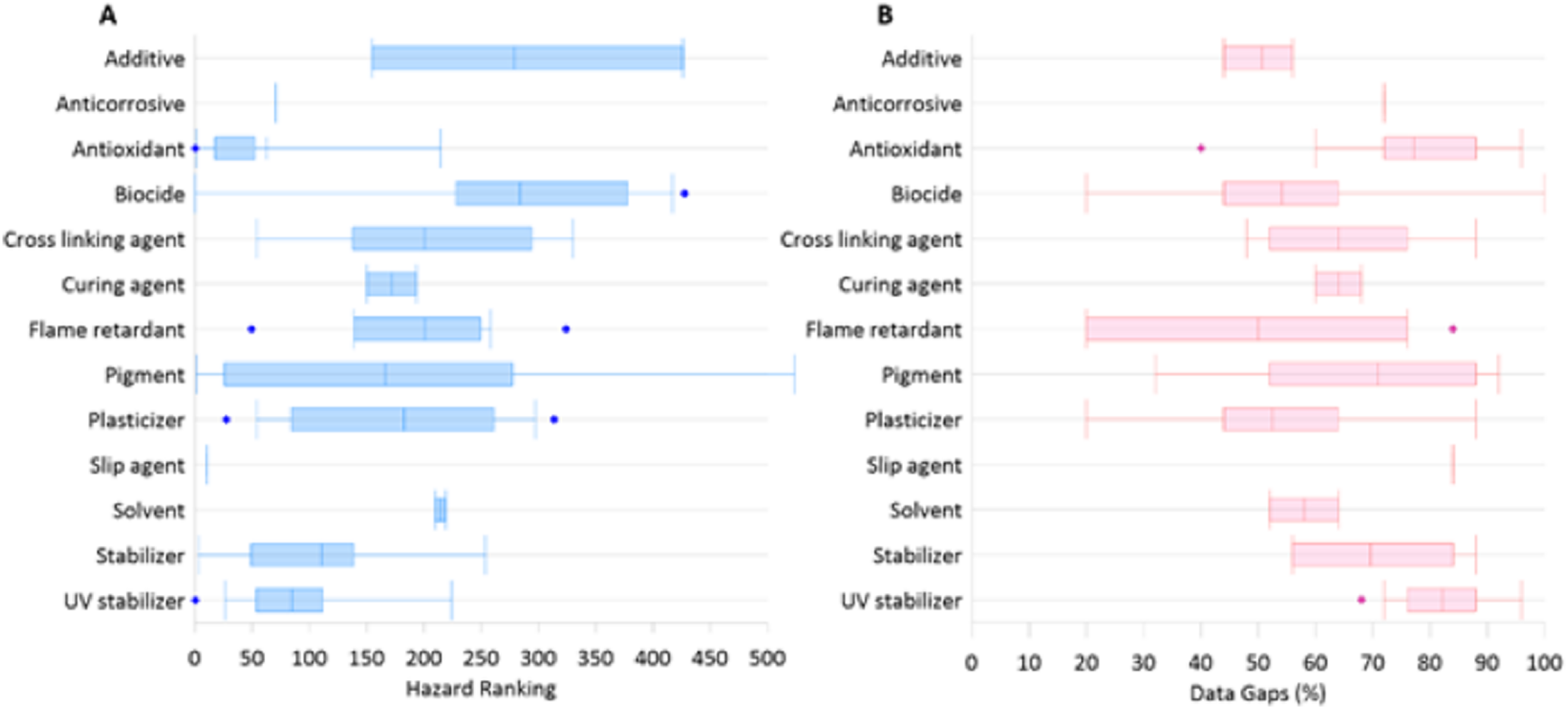
Rankings of plastic additives based on function. (A) Hazard rankings of based on additives key functional property, higher values indicate higher hazard ranking. (B) Overall data gaps associated with each additive.

In a similar fashion, we have reviewed the available literature and compiled a list of chemical additives commonly present in plastic and identified to be of potential emerging concern in New Zealand aquatic ecosystems (see Supplementary Material, Table S1). These ninety-five chemicals are grouped into 14 categories based on the primary functional property they provide in finished plastic materials and documented and ranked their potential hazard (integrated information on the toxicity, ecotoxicity, environmental fate, and physicochemical properties) using information provided by the Pharos project, a resource that compiles information from over 40 regulatory agencies worldwide (Fig. 2) (https://pharosproject.net/comparisons/320). This same database was also interrogated to identify data gaps (where data for specific hazard classifications are missing) for each group of additives.

**Figure 3:**
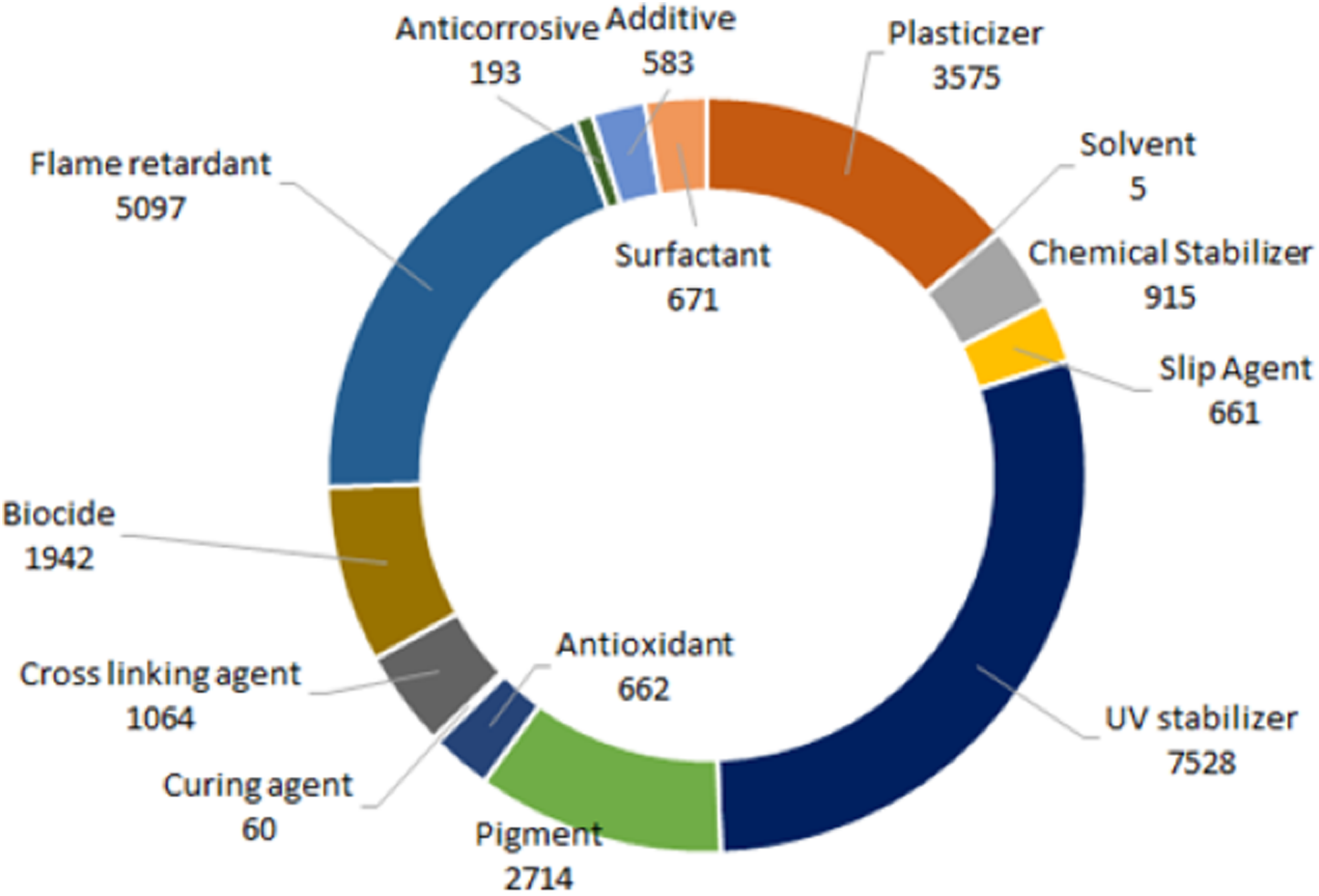
Total ecotox endpoints available. Summary of total ecotoxicology data available in regulatory databases for selected additives, classified by main function. Data consists of a wide array of endpoints ranging from gene expression to reproductive success. Information was compiled from US EPA comptox, EPA Ecotox Knowledgebase, Pesticide Action Network Framework, ECHA substance fact sheets, Environment Canada, and NZ EPA.

To further elucidate the ecotoxicological assessments and data that has been reported for each additive on the list, publicly available ecotox information for each additive was identified and compiled from the USEPA comptox, EPA Ecotox Knowledgebase, Pesticide Action Network Framework, ECHA substance fact sheets, Environment Canada, and NZ Environmental Protection Agency (Fig. 3). Ecotox data ranged from a wide of endpoints ranging from gene expression to population changes.

This assessment clearly demonstrates that previous ecotoxicological investigations have focused on four main groups of additives: (i) UV stabilizers, (ii) flame retardants, (iii) plasticizers and (iv) pigments. Over half of the ecotoxicological endpoints for the plastic additives we assessed were reported for only five additives: bisphenol A, dibutyl phthalate, tetrabromobisphenol A, di(2-ethylhexyl) phthalate and tributyltin oxide. The information provided by our short list of additives is limited in scope compared to the huge number of additive chemicals that are available for use in plastics. The fact that only 18 of these same additives are included in the ECHA’s plastic additive list provides some context for the ecotoxicity data that is currently available, and more importantly, where it is lacking.

**Figure 4:**
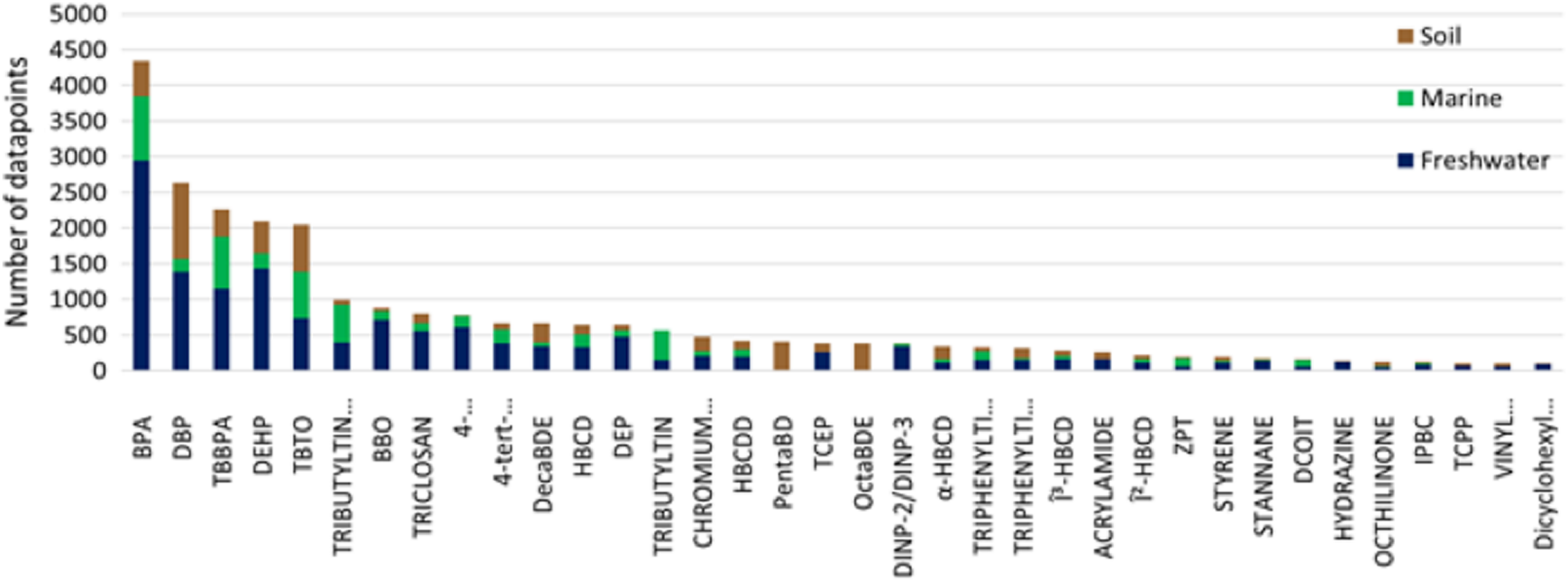
Data available for plastics additives based on environmental compartment. Chemical additives commonly used as plastic additives with over a 100 ecotox data points available. Data is partitioned into three environmental compartments: freshwater, marine and soil.

Plastic additives classified as POPs have been extensively assessed for their toxicity which reflects the large number of ecotoxicological data points reported (OECD, 2019a). Many other additive chemicals are semi-volatile organic chemicals that have found broad applications over a wide range of industries for several decades. Consequently, these chemicals have also been subject to intensive assessment of their toxicity and environmental impact, with the result they also have a similarly high number of ecotoxicological data points (i.e., phthalates, UV stabilizers, and flame retardants). To elucidate general trends on ecotoxicity data for comprehensively tested chemicals we took the additives from our data set with more than 100 reported ecotoxicological data points (obtained from the previously described sources) and classified them based on environmental compartment the ecotoxicological data were derived (Fig. 4).

Unsurprisingly, most of the available ecotoxicological information was derived for freshwater ecosystems, which is in largely due to the prevalence of assays using freshwater organism models and demonstrate regulatory compliance. There is less soil ecotoxicity information compared to freshwater, which reflects the complexity of conducting toxicity testing in the soil matrix. The least amount of ecotoxicity information for the selected additives was for the marine environment. Only tributyltin, zinc pyrithione and 3(2H)-isothiazolone,4,5-dichloro-2-octyl- had more data points available for marine organisms compared to freshwater and soil, probably due to their extensive applications in marine antifouling paints. Many of these additives may cause toxicity at relatively low concentrations in aquatic ecosystems (below 1 mg/L) and potentially pose a risk to exposed biota. Given the uncertainties surrounding MNPs as a potential vector for the source, transportation, and delivery of these chemical additives in the environment, further investigations are warranted. Considering the select additives’ soil adsorption coefficient (Koc) and octanol partition coefficient (Kow) many of these additives are likely to impact terrestrial environments and considerations need to be as to identifying which environmental compartments are likely to be impacted (see Supplemental Material, Table S3).

### Micro(nano)plastic effect vs additive effect

In general plastic polymers are considered to have low bioavailability due to their inherently high molecular weight limiting environmental transport and passage across biological membranes prior to environmental fragmentation (ECHA, 2012). Despite this, some plastics including polyacrylates, polyurethanes, polyvinyls, expanded polystyrene, epoxy resins and polyacrylonitriles are recognized to be potentially hazardous (OECD, 2018) due to the presence of unreacted monomers and oligomers which have been demonstrated to have carcinogenic or mutagenic effect. Presently, a correlation is yet to be made but there is a strong relationship between the quantity of unreacted monomers and oligomers in plastic polymers and the associated hazard (Hermabessiere et al., 2017).

Migration of additives into surrounding media is a diffusion controlled process and as such is primarily contingent upon: (i) the porosity of the polymer structure/thickness, (ii) molecular weight of the additives, (iii) hydrophobicity of the additives and (iv) the properties of the surrounding media (Teuten et al., 2009). The need to discriminate between the ecotoxicity of MNPs themselves and their associated additives is highlighted by research demonstrating additives may rapidly diffuse from plastic polymers into surrounding biological tissue (Booth et al., 2019). It is important to consider that additives are present in plastic polymers as mixtures, and as such exposure to MNP particles could potentially lead to multiple mechanisms of toxicity in receptor organisms. For example, the presence of biocidal additives in MNPs may prevent microorganisms from utilizing other chemical additives as a source of energy and thus preventing degradation, resulting in organisms being exposed to unusually high concentrations of residues than would otherwise be the case (Hansen, Nilsson & Slot Ravnholt Vium, 2014). The assessment of additive toxicity vs MNP toxicity is an emerging research topic with increasing interest but little standardization, presenting a significant barrier to establishing concise conclusions on the potential hazards they present in the environment (Beiras, Tato & López-Ibáñez, 2019; Luo et al., 2019a, 2019b; Oliviero et al., 2019; Capolupo et al., 2020). This issue is exacerbated by the fact that degradation of plastic through weathering and ageing makes ecotoxicity assessment challenging as the number of potentially leachable chemical additives and polymer degradation products can be likened to a veritable cocktail of chemical toxicants and stressors (Hansen et al., 2013; Fred-Ahmadu et al., 2020).

Weathering of plastics can occur through physical or chemical degradation with the predominant mechanisms being photolytic, thermolytic, oxidative, ozone-induced, hydrolytic and biological (Singh & Sharma, 2008; Webb et al., 2013). Functionally, degradation of plastics can begin with any of these processes but it is commonly initiated by UV-radiation inducing oxidation, of the polymer chains leading to structural degradation and ultimately fragmentation which can result in bioaccumulation as smaller particles are more likely to be taken up by organisms in the ecosystem. Different plastic polymers exhibit different degradation patterns in the environment which further complicates the hazard characterization of both the plastic polymers as well as the associated additives (Webb et al., 2013). Ecotoxicological assessment requires the ability to discriminate between the physical effects of MNPs from those associated with the degradation of these particles, and there is a growing interest in discriminating between the MNPs and the additives they contain. As MNPs can pose adverse biological effects as particles and through chemical additives, it is crucial to investigate both modes of toxicity. Presently there are significant gaps in knowledge surrounding comparative studies on the ecotoxicity of different polymer types and the effects of subsequent weathering.

Weathering processes can both increase and decrease the adsorption/absorption capacity of MNPs for ambient contaminants and alter their mobility through environmental compartments (Rist & Hartmann, 2018; Liu et al., 2019). There is also the consideration of whether or not digestive processes within organisms that ingest MNPs can increase the rate or magnitude of chemical additives from plastic particles (Koelmans, Besseling & Foekema, 2014).

Function of additives also needs to be taken into consideration as, for instance, oxo-degradative substances incorporated into plastics facilitates fragmentation making it difficult to estimate the relative stability of oxo-modified polymers from their respective unadulterated form (OECD, 2018). While many studies investigating the effects of plastic leachates have been conducted, there is a paucity of comparative studies that discriminate between the impact of MNP degradation and the concomitant release of additives. Such discriminative ecotoxicological investigations are essential for determining if: (i) MNPs are themselves a hazard; (ii) plastic degradation results in the release of toxic compounds or (iii) the increased release of additives from MNPs raises ecotoxicological risks.

### Future research needs for micro(nano)plastic ecotoxicity

MNPs have been identified as emerging contaminants of concern but the lack of standardization in the methods used for ecotoxicity testing does not allow for meaningful comparisons between studies. Current limitations in characterizing plastic (e.g., polymer types, sizes, morphology, ageing/weathering…) combined with limitations associated with sampling and isolation procedures from environmental and biological tissue samples highlights the need for an improved classification for plastic toxicity as a generic definitions are insufficient as size and shape of particles can alter biological uptake and tissue localization (Regoli et al., 2017; Bessa et al., 2019; Cole et al., 2019; Hartmann et al., 2019). There is also a need for improved methods to characterize the leaching of chemical additives and environmental contaminants from MNPs into relevant environmental media, followed by their bioavailability and uptake into biota subsequently exposed to them. Additional consideration needs to be given to providing improved descriptions of the properties of polymers in MNPs. Similarly, specific information on chemical additives including their percentage composition in plastics, and resulting MNPs, and relevant physicochemical properties for predicting migration into surrounding media (e.g. solubility, size of chemical, vapor pressure, boiling point…) through the whole life cycle of MNPs is necessary to predict their release into the environment during the whole-life cycle of plastic materials (Hansen, Nilsson & Slot Ravnholt Vium, 2014). As part of this assessment, there is a need to consider the solubility of the additives as it is a major contributor to which environment compartment they are likely to impact. Hydrophobic additives are not all soluble in the same chemical solvent which limits the ability to establish meaningful comparisons between additives within the same class (Hansen et al., 2013).

There is an urgent need for representative plastic reference materials to evaluate ecotoxicity, including in combination with representative high purity additives for establishing clear regulatory guidelines for MNPs (Rist & Hartmann, 2018). MNP toxicity is a broad research topic and as such any reference materials should represent (i) particles at the micro and nano scales and (ii) environmentally representative particles that including naturally weathered compounds. Many previous ecotoxicological studies have used pristine, uniform particles of similar size, shape, and polymer type for expedience and simplicity of experimental design and implementation. Too often these experiments do not represent real world exposures and their outcomes rarely add to our understanding the hazard of MNPs in the environment.

Investigating the effects of MNPs in a natural setting is next to impossible, so it is essential to conduct laboratory assessments using environmentally representative materials that take account of natural weathering processes (JPI Oceans, 2018). This has led to the increasing requirement for environmentally relevant MNPs or reference materials that are representative of various polymer types and plastic materials at different stages of environmental weathering, together with improved protocols for characterizing their release of additives (Jahnke et al., 2017).

Clear guidelines and procedures are also needed to prepare test suspensions of MNPs for ecotoxicity assessment. Much can be gained by referring to guidelines and procedures previously developed to assess the ecotoxicity of nanomaterials. Recording and reporting key information such as the size distribution, density, functional surface charge and functional group composition is essential to facilitate the development of harmonized testing approaches (Connors, Dyer & Belanger, 2017; Rist & Hartmann, 2018). This is important as the methods used to prepare homogenous test suspensions of MNPs in aqueous media or incorporate them into soil and sediment for toxicity assessment, can influence their subsequent bioavailability and toxicity. This can be very challenging. For example, the buoyancy of MNPs in aqueous media is dependent on the density of polymer, porosity, size, and potential surface charges. Improved characterization of MNPs test suspensions has been identified as a research need for all environmental matrices but currently no clear protocols exist (Connors, Dyer & Belanger, 2017; JPI Oceans, 2019). Various methods are applied to report the concentration of MNPs including total mass, particle number, and total surface area. This disparity limits the ability to establish clear comparisons between studies and illustrates the need to establish clear guidelines for reporting concentrations of MNPs in different environmental media (Connors, Dyer & Belanger, 2017).

The standardization of tests for the characterization of MNPs comes with the prerequisite of a standardization of definitions, and common procedures for collating and reporting key experimental criteria and methodology including information regarding sample collection, storage and preservation, extraction from environmental media (including tissue samples), chemical compositions (including additives), and quantification of MNPs (Murphy, 2017). Appropriate definitions for the size of MNPs and other key descriptions that are necessary to establish inter and intra laboratory comparisons for MNPs ecotoxicity are currently being discussed and debated (Frias & Nash, 2019). Clear guidelines for physicochemical characterization (inherent properties and properties in test media) also need to be generated to improve the comparability between ecotoxicological experiments to further elucidate how these properties impact environmental health (Mitrano & Wohlleben, 2020). Guidelines are currently being developed to harmonize these testing strategies for MNPs (Bessa et al., 2019).

It is essential to establish standard ecotoxicity testing that can reproducibly and reliably characterize hazards of MNPs to assess the environmental risks. Internationally recognized testing frameworks, such as OECD test guidelines and ISO standards, provide useful starting points. However, the experimental studies completed to date demonstrate standard toxicity assays (such as acute ecotoxicity assay) may not capture key aspects of MNP impact and alternative long-term exposure experiments measuring endpoints at lower levels of biological organization may be necessary (Regoli et al., 2017). Assays that target specific mechanisms of toxicity as well as multigenerational testing may be necessary to fully characterize ecosystem effects. Integrating biomarkers into the assessment of MNPs toxicity will assist the identification of a broader range of potential pathways of toxicity and facilitate the development of toxicodynamic assessments for MNPs (Murphy, 2017). The incorporation of low cost, high throughput, assay formats that test a wide array of different biological endpoints in reduced timeframes will also facilitate this approach and assist researchers to differentiate between the toxic effect of plastic polymers and MNPs themselves, and the toxicity of chemical additives. This is particularly true for characterizing the localization of MNPs and subsequent translocation of additives into different types of biological tissues.

When considering the hazards of MNPs, it is necessary to consider how MNPs composed of different polymer types will migrate to and accumulate within different environmental compartments. For example, MNPs composed of polymers with a density greater than 1 g/mL sink and accumulate in bottom sediments where benthic organisms will be the main receptor species. MNPs with a density less than or equal to one 1g/mL will remain buoyant in the water column, be transported greater distances, and accumulate along shoreline of lakes and banks of rivers and streams, and the tidal line of beaches, where they are subjected to weathering from abrasion and exposure to sunlight. MNPs that are buoyant in the water column accumulate biomass that increases their density and may ultimately lead to them sinking and accumulating in sediments. It is however important to note that biofouling and aggregation of MNPs will likely occur for all polymers and the particles will eventually accumulate in the sediment of receiving environments.

This suggests the need to consider using multiple organisms to characterize the broader range of exposure and impacts associated with MNPs in different environmental compartments, and within trophic structures in the same. While some of the established test species used for ecotoxicity assessments will be suitable for assessing the toxicity of MNPs it is envisaged there will be a need to validate additional test species (Murphy, 2017; Rist & Hartmann, 2018). This can include organisms that have socio-economic relevance and significant commercial value, species representing an expanded range of taxa, and species that fill specific niches in ecosystems. To support a comprehensive hazard assessment there is a need for improved techniques to assess the bioavailability of MNPs and associated additives, distribution and localization within biota, migration of additives across biological barriers, and characterization of submicron/nanosized plastic particles and fibers in biota.

### Regulatory perspectives on micro(nano)plastics

Effective management of plastic waste requires effective governance and mitigation options. One of the major points of release of MNPs is through wastewater effluents which is major focal point release into marine environments (Edo et al., 2020; Franco et al., 2021; Naji et al., 2021). Management of wastes also requires identification and prioritization of chemical additives that may pose a significant hazard to the environment and exposed biota prior to release. The implementation of safe(r)-by-design (SbD) strategies can assist the chemical and plastics industries to manufacture products that cause less environmental harm through the use of alternative additives with reduced toxicity, and/or additives with increased specificity that enables lower concentrations to be used to achieve the desired properties (Cobaleda-Siles et al., 2017). This can be achieved by targeting chemicals exhibiting physicochemical properties (molecular weight, solubility, boiling point) identified to minimize their leaching and migration from plastics during their whole life cycle. In the context of SbD, there needs to be serious consideration of intended aims for creating “safer” plastics. For example, using additives with higher water solubilities increases their toxic potential to organisms in the water column but also increases the dispersibility of the additive, whereas, less soluble additives are likely to leach out of plastic in reduced quantities but have an increased potential for bioavailability and bioaccumulation.

### European perspectives

Under the European Green Deal, addressing environmental hazards associated with plastic wastes and the MNPs they produce is one of the European Commission’s key priorities (European Commission, 2019). The Commission envisages focused actions to reduce the emission of MNPs resulting from the production of primary MNPs, and from their intentional incorporation into other manufactured products. In 2019, the European Chemical Agency (ECHA) proposed a restriction on the intentional uses of MNPs in products placed on the EU/EEA market to avoid or reduce their release to the environment. It is estimated the proposed restriction, recently adopted (June 2020) by the ECHA’s Committee for Risk assessment (RAC), will reduce the release of MNPs by more than 90%. It is important to note however that this pertains to primary plastics created at the micro and nanoscale rather than secondary degradation of plastics, which are likely the majority of MNPs found in the environment.

As previously mentioned, a joint project focused on characterizing the uses of plastic additives, and the extent in which the additives may be released from plastic articles, was launched by ECHA and the 21 industry sector organizations. This project has reviewed over 400 high volume plastic additives used in the EU with the aim of characterizing the scope of MNPs that are an environmental issue. Moving forward, public-private partnerships with the appropriate industry groups are likely to be required to identify and compile the information required to establish a meaningful and enduring governance strategy for plastic additives.

A Science Advice for Policy by European Academies (SAPEA) consortium concluded, the available scientific knowledge on MNPs and their potential effects on the environment remain uncertain and further research effort is required to cover the complexity of this challenge (SAPEA, 2019). A knowledge gap identified by SAPEA was the lack of information regarding the characterization of nanosized fraction of MNP fragments present in the environment and the associated chemical additives and their potential release into the environment and biota. Many previous studies have emphasized the need for developing and/or improving international harmonization of methods used to assess exposure, fate and effect of MNPs on biota from different environmental compartments (water, sediment and soil). Meanwhile, very little is known about the occurrence and characteristics of nanosize fragments of plastics compared to MNPs as very few sampling and analysis methods have been reported, they are complex, and far from becoming standardized. To reduce the existing methodological and data gaps, the European Commission’s Join Research Center (JRC) has announced several calls on the standardization and harmonization of sampling and analytical measurement protocols for micro and nano plastics along with validated methods, reference materials and initiatives to assess the occurrence of MNPs in wastewater.

In an ecotoxicological perspective, SAPEA have reported limitations of recent studies and highlighted that effort should be made in the future to investigate environmental effects. Whatever the environmental compartment, questions remain about the long-term effects of MNPs retained by organisms. Additional effort is required to improve our knowledge on the nanosized fragment of plastics as reductions in size can enhance both accumulation in biological and potential ecotoxicity.

### New Zealand perspectives

Investigations into the environmental effects of MNPs is sparse in New Zealand but there is increasing concern regarding the potential threat MNPs present to the countries’ “green” reputation, unique ecosystems, primary industry, and the clean reputation of the countries’ export focused economy. Limited research has previously been conducted to characterize the distribution and magnitude of MNP contamination in New Zealand’s environment, but research seems to suggest it is similar in scale to what has been reported in other industrialized nations (Clunies-Ross et al., 2016; Dikareva & Simon, 2019; Bridson et al., 2020; Mora-Teddy & Matthaei, 2020). A majority of the studies conducted in New Zealand have focused on large cities which may not be representative of the country as a whole (Webb et al., 2019). There is also a lack of information on MNPs in soils which has led to concerns over the potential implications of chemicals transferring to agricultural products and potential commercial implications towards New Zealand primary industries’ that benefit from the perception held in other countries that NZ is relatively free of contamination. This is particularly poignant as the country as a growing interest in the use of biosolids which can have high concentrations of MNPs (Okoffo et al., 2020). Research of MNPs in NZ would benefit from leveraging off the country’s inherent topography and adopting a mountain to-sea total catchment approach that integrates multiple environmental compartments including freshwater and sediment, estuarine and intertidal, and inshore coastal ecosystems. This includes characterizing regions of the country that have had next to no anthropogenic interactions to identify whether MNPs are prevalent in the nation’s pristine environments.

In New Zealand’s there is an inclination to use local, native species in ecotoxicity rather than internationally recognized, standard species for the characterization of environmental hazards. This also includes using species with cultural relevance, or taonga species, and those of commercial value, for example the NZ green lipped mussel. In the context of environmental concerns, information on the use and release of plastics intentionally created at the micro and nano scale is limited and information regarding the presence of primary MNP particles needs to be investigated. As with other countries, initiatives also need to focus on the degradation of macroplastics in the environment and the release of associated chemicals and New Zealand initiatives into MNP risks will need to emphasize preventative approaches that limit the potential release.

## Conclusions

The risk characterization of MNPs is a nuanced, multifaceted issue. Much of the research on MNP toxicity has primarily focused on the toxicity of the particles themselves but there is a recognized need to investigate and characterize the potential associated environmental risks of the varied additives inherently present in plastic (Gunaalan, Fabbri & Capolupo, 2020). The concentration of additives in plastic polymers is dependent on function but up to 70% of weight has been previously reported, making plastic additives a potentially underrepresented hazard (Hahladakis et al., 2018). To appropriately assess these risks, key research gaps associated with chemical additives need to be addressed including:

- Improved characterization of MNPs as potential vectors for additive release and their subsequent accumulation in abiotic and biotic media
- Ranking the toxicity and subsequent risk of chemical additives released from MNPs against that of other anthropogenic contaminants they accumulate from the environment
- Characterization of the relationship between polymer types, additives, and their toxicity profiles to determine if different types of MNPs alter the potential risk of these chemicals
- Investigating and assessing the risk of MNPs as contaminant mixtures rather than single contaminants, with emphasis being placed on linking key physicochemical properties of additives to mechanistic responses
- Establish testing approaches and representative species that integrate the risk of MNPs and chemical additives across multiple ecosystems, such as the mountain-to-sea approach, to further elucidate ecotoxicity along a particle’s lifecycle
- Application of more sensitive, sublethal endpoints that can potentially link mechanistic responses of MNP polymers and associate additives to adverse outcomes at higher levels of biological organization including populations and ecosystem functions

Differences in polymer types and density also suggest that different environmental compartments will likely be impacted by different chemicals. Additives also have varying solubilities, with many of them having low solubilities in aqueous media. Testing on several organisms, using multiple levels of biological organization, will be necessary to fully characterize the toxicity of MNPs. It is also important to consider that emphasis is being placed on developing (bio)degradable plastics/polymers, which can change the release profiles of potentially harmful additives. Clear guidelines and standardized methods for the collection MNPs from the environment and assessment of ecotoxicity are also needed to establish meaningful comparisons between different polymer types and the respective release of additives. These links also need to further investigate the relationship between additive release and micro/nano interface as the size of particles is known to alter the physicochemical properties of MNPs which in turn affects their toxicity. Establishing benchmark MNP testing materials, as standardized comparators, would assist the development of robust guidelines for assessing the environmental risk of MNPs.

The ecotoxicological data for many additives currently incorporated within plastic is limited and even less information exists regarding their presence in the environment. This knowledge gap could be addressed with the collaboration and support of the polymer industry which has previously been reluctant to publicly release information on the types and composition of additives present in the masterbatches they produce and provide to manufacturers of plastics.

Research into additive ecotoxicity would also benefit from the implementation of a SbD approach to characterize environmental hazards as it is unlikely the use of plastic additives will cease. Implementation of SbD initiatives that include the characterization of the ecotoxic potential of plastic additives would help guide the polymer industry to identify potential alternatives with reduced environmental risk prior to their entry into the marketplace. Ultimately, however, the most effective method of reducing plastic risks is through the minimizing outflow of plastic wastes into the environment through the application of a circular economy.

## Supplemental Information

10.7717/peerj.11300/supp-1 Supplemental Information 1 Plastic additives used in study. List of plastic additives compiled from the Pharos project to be of emerging concern in New Zealand waters. Additives are classified by key functional properties.

10.7717/peerj.11300/supp-2 Supplemental Information 2 Ecotoxicity thresholds for plastic additives. Publicly available ecotoxicity data public available from ECHA substance factsheets, NZ EPA, pesticideinfo.org, Enviroment Canada and the US EPA Ecotox Knowledgebase. * indicate that values where the lowest reported value was taken.

10.7717/peerj.11300/supp-3 Supplemental Information 3 Total ecotoxicity data available paritioned by environmental compartment. Compiled ecotoxicity data partitioned by primary environmental compartments. Data was compile from USEPAcomptox, ECHA substance factsheets, NZ EPA, pesticideinfo.org, Environment Canada and the US EPA Ecotox Knowledgebase

## Additional Information and Declarations

### Data Availability

The following information was supplied regarding data availability: Data are available in the Supplemental Files.
