# Supplementary material for: Plastic additives: challenges in ecotox hazard assessment
Source: PeerJ. 2021 Apr 16;9:e11300. doi: 10.7717/peerj.11300 (PMC8054737; doi:10.7717/peerj.11300)
Supplement: Supplemental Information 1 — List of plastic additives compiled from the Pharos project to be of emerging concern in New Zealand waters. Additives are classified by key functional properties. [file peerj-09-11300-s001.docx]

| Name | CAS | Key Functional Property |
| --- | --- | --- |
| 3(2H)-ISOTHIAZOLONE, 4,5-DICHLORO-2-OCTYL- | 64359-81-5 | Biocide |
| DECABROMODIPHENYLETHANE (DBDPE) | 84852-53-9 | Flame retardant |
| ZINC PYRITHIONE (ZPT) | 13463-41-7 | Anticorrosive |
| TRIS(2-CHLOROETHYL) PHOSPHATE (TCEP) | 115-96-8 | Flame retardant |
| 3-IODO-2-PROPYNYL BUTYLCARBAMATE | 55406-53-6 | Biocide |
| BENZOTRIAZOLE | 95-14-7 | Anticorrosive |
| BENZIMIDAZOLE | 51-17-2 | Biocide |
| 2-ETHYL-1-HEXANOL | 104-76-7 | Plasticizer |
| Stannane, fluorotriphenyl- | 379-52-2 | Biocide |
| 2-(2H-BENZOTRIAZOL-2-YL)-4,6-BIS(1-METHYL-1-PHENYLETHYL)PHENOL | 70321-86-7 | UV stabilizer |
| 3,3'-THIOBISPROPANOIC ACID, DITRIDECYL ESTER | 10595-72-9 | antioxidant |
| Oleyl amide | 301-02-0 | Slip agent |
| Dialkyl(C7-11-branched and linear) Phthalate (DHNUP) | 68515-42-4 | Cross linking agent |
| HEXABROMOCYCLODODECANE (HBCDD) | 3194-55-6 | Flame retardant |
| OCTABROMODIPHENYL ETHER (OCTABDE) | 32536-52-0 | Flame retardant |
| BETA-HEXABROMOCYCLODODECANE (Î²-HBCD) | 134237-51-7 | Flame retardant |
| STYRENE | 100-42-5 | Plasticizer |
| STANNANE, ACETOXYTRIPHENYL | 900-95-8 | Biocide |
| HYDRAZINE | 302-01-2 | Additive |
| OCTHILINONE | 26530-20-1 | Biocide |
| VINYL CHLORIDE (VCM) | 75-01-4 | Plasticizer |
| HYDRAZINE, MONOHYDRATE | 7803-57-8 | Additive |
| Dimethoxyethyl phthalate (DEMP) | 117-82-8 | Plasticizer |
| Cadmium selenide | 1306-24-7 | Pigment |
| CHLORINATED PARAFINS (AVERAGE CHAIN LENGTH, C12; APPROXIMATELY 60 PERCENT CHLORINE BY WEIGHT) | 108171-26-2 | Flame retardant |
| Hexanedioic acid, diheptyl ester | 14697-48-4 | Antioxidant |
| Phenol, 2-(2H-benzotriazol-2-yl)-4-(1,1-dimethylethyl)- | 3147-76-0 | UV stabilizer |
| Benzenesulfonic acid, 4-[[1-[[(2-chlorophenyl)amino] carbonyl]-2-oxopropyl]azo]-3-nitro-, calcium salt (2:1) | 71832-85-4 | Pigment |
| DIISOHEPTYL PHTHALATE (DIHP) | 71888-89-6 | Plasticizer |
| allyltriphenyl stannane | 76-63-1 | Biocide |
| TRIBUTYLTIN | 688-73-3 | Flame retardant |
| PENTABROMODIPHENYL ETHER (PENTABDE) | 32534-81-9 | Flame retardant |
| Benzophenone | 119-61-9 | Additive |
| NONYLPHENOL PHOSPHITE (3:1) | 26523-78-4 | Stabilizer |
| BUMETRIZOLE | '3896-11-5 | UV stabilizer |
| 1,3,5-TRIS-[(2S AND 2R)-2,3-EPOXYPROPYL]-1,3,5-TRIAZINE-2,4,6-(1H,3H,5H)-TRIONE | 59653-74-6 | Cross linking agent |
| BUTYLATED HYDROXYANISOLE (BHA) | 25013-16-5 | Stabilizer |
| 1,3,5-TRIAZINE-2,4,6(1H,3H,5H)-TRIONE,1,3,5-TRIS((4-(1,1-DIMETHYLETHYL)-3-HYDROXY-2,6-DIMETHYLPHENYL)METHYL)- | 40601-76-1 | Plasticizer |
| TETRABROMOBISPHENOL A (TBBPA) | 79-94-7 | Flame retardant |
| TRIBUTYLTIN CHLORIDE | 1461-22-9 | Biocide |
| DIETHYL PHTHALATE (DEP) | 84-66-2 | Plasticizer |
| ALPHA-HEXABROMOCYCLODODECANE (&alpha,-HBCD) | 134237-50-6 | Flame retardant |
| TRIPHENYLTIN HYDROXIDE | 76-87-9 | Biocide |
| ACRYLAMIDE | 79-06-1 | Cross linking agent |
| Dicyclohexyl phthalate | 84-61-7 | Plasticizer |
| TRIBUTYLTIN METHACRYLATE | 2155-70-6 | Biocide |
| DIISOBUTYL PHTHALATE (DIBP) | 84-69-5 | Flame retardant |
| Butylated hydroxytoluene | 128-37-0 | Flame retardant |
| 2,2'-BIS-6-TERC.BUTYL-P-KRESYLMETHAN | 119-47-1 | Flame retardant |
| 2-(2'-HYDROXY-5'-METHYLPHENYL)BENZOTRIAZOLE | 2440-22-4 | Flame retardant |
| 2-benzotriazol-2-yl-4,6-di-tert-butylphenol | 3846-71-7 | Flame retardant |
| 6,6'-di-tert-butyl-4,4'-diethyl-2,2'-methylenediphenol | 88-24-4 | Flame retardant |
| 5,12-DIHYDROQUINO(2,3-B)ACRIDINE-7,14-DIONE | 1047-16-1 | Flame retardant |
| DIBUTYL PHTHALATE (DBP) | 84-74-2 | Plasticizer |
| 10,10'-BIS(PHENOXYARSINYL) OXIDE | 58-36-6 | Biocide |
| 4,4'-METHYLENEBIS(2-CHLOROANILINE) | 101-14-4 | Curing agent |
| PROPYL 3,4,5-TRIHYDROXYBENZOATE | 121-79-9 | Antioxidant |
| 4,4'-METHYLENE DIANILINE (MDA) | 101-77-9 | Cross linking agent |
| DECANEDIOIC ACID, BIS(1,2,2,6,6-PENTAMETHYL-4-PIPERIDINYL) ESTER; | 41556-26-7 | UV stabilizer |
| TRIBUTYLTIN OXIDE (TBTO) | 56-35-9 | Biocide |
| TRICLOSAN | 3380-34-5 | Biocide |
| 4-Nonylphenol (branched) | 84852-15-3 | Plasticizer |
| CHROMIUM (VI) OXIDE | 1333-82-0 | Pigment |
| TRIPHENYLTIN CHLORIDE | 639-58-7 | Biocide |
| GAMMA-HEXABROMOCYCLODODECANE (Î³-HBCD) | 134237-52-8 | Flame retardant |
| TRIS(1-CHLORO-2-PROPYL)PHOSPHATE (TCPP, TMCP) | 13674-84-5 | Flame retardant |
| 2-METHOXYETHANOL | 109-86-4 | Solvent |
| 2-Ethylhexanoic acid | 149-57-5 | Solvent |
| SHORT CHAIN CHLORINATED PARAFFINS (SCCP), C10-13 | 85535-84-8 | Plasticizer |
| Tributyltin methacrylate, methyl methacrylate polymer | 26354-18-7 | Biocide |
| Tris(2,4-di-tert-butylphenyl) phosphite | 31570-04-4 | Antioxidant |
| 1-[4-(1,1-dimethylethyl)phenyl]-3-(4-methoxyphenyl)propane-1,3-dione | 70356-09-1 | UV stabilizer |
| ANOX 20 | 6683-19-8 | Antioxidant |
| (Z)-13-DOCOSENAMIDE | 112-84-5 | Stabilizer |
| TRIGLYCIDYL ISOCYANURATE (TGIC) | 2451-62-9 | Cross linking agent |
| QUINO 2,3-B ACRIDINE-7,14-DIONE, 2,9-DICHLORO-5,12-DIHYDRO- | 3089-17-6 | Pigment |
| 2-(2H-benzotriazol-2-yl)-4-(tert-butyl)-6-(sec-butyl)phenol | 36437-37-3 | UV stabilizer |
| DECABROMODIPHENYL ETHER (DecaBDE) | 1163-19-5 | Flame retardant |
| Benzoic acid, 2-[[3-[[(2,3-dihydro-2-oxo- 1H-benzimidazol-5-yl)amino]carbonyl]-2-hydroxy-1 -naphthalenyl]azo]-, butyl ester | 31778-10-6 | Pigment |
| 2-(2'-HYDROXY-5'-T-OCTYLPHENYL)BENZOTRIAZOLE | 3147-75-9 | UV stabilizer |
| BUTYL BENZYL PHTHALATE (BBP) | 85-68-7 | Plasticizer |
| CADMIUM SULFIDE | 1306-23-6 | Pigment |
| 2-tert-butylhydroquinone | 1948-33-0 | Antioxidant |
| 2,2,6,6-tetramethylpiperidine | 768-66-1 | Antioxidant |
| 4-tert-Octylphenol | 140-66-9 | Plasticizer |
| BISPHENOL A (BPA) | 80-05-7 | Flame retardant |
| DI(2-ETHYLHEXYL)PHTHALATE (DEHP) | 117-81-7 | Plasticizer |
| HEXABROMOCYCLODODECANE (HBCD, HBCDD) | 25637-99-4 | Flame retardant |
| Diisononyl phthalate (DINP-2 or DINP-3, mixture of isomers as manufactured) | 28553-12-0 | Plasticizer |
| DI(2-ETHYLHEXYL)ADIPATE (DEHA) | 103-23-1 | Plasticizer |
| BISPHENOL A DIGLYCIDYL ETHER (BADGE) | 1675-54-3 | Cross linking agent |
| Formaldehyde, polymer with benzenamine | 25214-70-4 | Curing agent |
| DI-N-PENTYL PHTHALATE (DNPP) | 131-18-0 | Plasticizer |
| DODECYL 3,4,5-TRIHYDROXYBENZOATE | 1166-52-5 | Antioxidant |
| phenol, 2-(2H-benzotriazol-2-yl)-6-dodecyl-4-methyl- | 23328-53-2 | UV stabilizer |
| PHENOL, 2-(5-CHLORO-2H-BENZOTRIAZOL-2-YL)-4,6-BIS(1,1-DIMETHYLETHYL)- | 3864-99-1 | UV stabilizer |
