# Supplementary material for: Plastic additives: challenges in ecotox hazard assessment
Source: PeerJ. 2021 Apr 16;9:e11300. doi: 10.7717/peerj.11300 (PMC8054737; doi:10.7717/peerj.11300)
Supplement: Supplemental Information 3 — Compiled ecotoxicity data partitioned by primary environmental compartments. Data was compile from USEPAcomptox, ECHA substance factsheets, NZ EPA, pesticideinfo.org, Environment Canada and the US EPA Ecotox Knowledgebase [file peerj-09-11300-s003.docx]

| Chemical | Freshwater | Marine | Soil | Total |
| --- | --- | --- | --- | --- |
| BPA | 2946 | 905 | 497 | 4348 |
| DBP | 1391 | 173 | 1074 | 2638 |
| TBBPA | 1148 | 728 | 387 | 2263 |
| DEHP | 1436 | 211 | 445 | 2092 |
| TBTO | 730 | 651 | 666 | 2047 |
| TRIBUTYLTIN CHLORIDE | 400 | 527 | 61 | 988 |
| BBO | 713 | 117 | 50 | 880 |
| TRICLOSAN | 549 | 113 | 142 | 804 |
| 4-Nonylphenol (branched) | 605 | 162 | 6 | 773 |
| 4-tert-Octylphenol | 390 | 190 | 91 | 671 |
| DecaBDE | 335 | 62 | 264 | 661 |
| HBCD | 329 | 181 | 129 | 639 |
| DEP | 480 | 89 | 70 | 639 |
| TRIBUTYLTIN | 144 | 423 | 0 | 567 |
| CHROMIUM (VI) OXIDE | 201 | 67 | 207 | 475 |
| HBCDD | 191 | 103 | 120 | 414 |
| PentaBD | 9 | 0 | 401 | 410 |
| TCEP | 267 | 0 | 123 | 390 |
| OctaBDE | 3 | 0 | 379 | 382 |
| DINP-2/DINP-3 | 339 | 36 | 3 | 378 |
| α-HBCD | 113 | 49 | 184 | 346 |
| TRIPHENYLTIN CHLORIDE | 153 | 120 | 52 | 325 |
| TRIPHENYLTIN HYDROXIDE | 145 | 25 | 148 | 318 |
| Î³-HBCD | 158 | 51 | 78 | 287 |
| ACRYLAMIDE | 157 | 8 | 95 | 260 |
| Î²-HBCD | 112 | 50 | 50 | 212 |
| ZPT | 57 | 115 | 21 | 193 |
| STYRENE | 119 | 17 | 54 | 190 |
| STANNANE | 141 | 11 | 18 | 170 |
| DCOIT | 65 | 84 | 17 | 166 |
| HYDRAZINE | 122 | 10 | 2 | 134 |
| OCTHILINONE | 53 | 8 | 65 | 126 |
| IPBC | 83 | 19 | 22 | 124 |
| TCPP | 67 | 0 | 37 | 104 |
| VINYL CHLORIDE | 55 | 0 | 47 | 102 |
| Dicyclohexyl phthalate | 97 | 0 | 3 | 100 |
| Benzophenone | 71 | 0 | 3 | 74 |
| TRIBUTYLTIN METHACRYLATE | 10 | 49 | 11 | 70 |
| 2-METHOXYETHANOL | 36 | 3 | 23 | 62 |
| DECABROMODIPHENYLETHANE (DBDPE) | 50 | 2 | 3 | 55 |
| BUTYLATED HYDROXYANISOLE (BHA) | 23 | 0 | 31 | 54 |
| DIISOBUTYL PHTHALATE (DIBP) | 27 | 2 | 23 | 52 |
| BENZOTRIAZOLE | 42 | 1 | 7 | 50 |
| 2-Ethylhexanoic acid | 9 | 0 | 35 | 44 |
| BENZIMIDAZOLE | 9 | 0 | 34 | 43 |
| 2-ETHYL-1-HEXANOL | 20 | 1 | 19 | 40 |
| Butylated hydroxytoluene | 20 | 1 | 18 | 39 |
| DI(2-ETHYLHEXYL)ADIPATE (DEHA) | 22 | 0 | 14 | 36 |
| 10,10'-BIS(PHENOXYARSINYL) OXIDE | 14 | 10 | 7 | 31 |
| BISPHENOL A DIGLYCIDYL ETHER (BADGE) | 25 | 0 | 1 | 26 |
| HYDRAZINE, MONOHYDRATE | 19 | 0 | 0 | 19 |
| CADMIUM SULFIDE | 15 | 0 | 3 | 18 |
| 2-tert-butylhydroquinone | 9 | 0 | 8 | 17 |
| 4,4'-METHYLENEBIS(2-CHLOROANILINE) | 5 | 0 | 11 | 16 |
| SHORT CHAIN CHLORINATED PARAFFINS (SCCP), C10-13 | 8 | 1 | 7 | 16 |
| Tributyltin methacrylate, methyl methacrylate polymer | 3 | 12 | 0 | 15 |
| PROPYL 3,4,5-TRIHYDROXYBENZOATE | 4 | 0 | 10 | 14 |
| Dimethoxyethyl phthalate (DEMP) | 9 | 0 | 1 | 10 |
| Stannane, fluorotriphenyl- | 6 | 2 | 2 | 10 |
| 4,4'-METHYLENE DIANILINE (MDA) | 5 | 0 | 4 | 9 |
| 2,2'-BIS-6-TERC.BUTYL-P-KRESYLMETHAN | 4 | 0 | 3 | 7 |
| 2-(2'-HYDROXY-5'-METHYLPHENYL)BENZOTRIAZOLE | 3 | 0 | 3 | 6 |
| Formaldehyde, polymer with benzenamine | 4 | 0 | 2 | 6 |
| 2-benzotriazol-2-yl-4,6-di-tert-butylphenol | 5 | 0 | 1 | 6 |
| DECANEDIOIC ACID, BIS(1,2,2,6,6-PENTAMETHYL-4-PIPERIDINYL) ESTER; | 5 | 0 | 1 | 6 |
| NONYLPHENOL PHOSPHITE (3:1) | 4 | 0 | 1 | 5 |
| Tris(2,4-di-tert-butylphenyl) phosphite | 2 | 0 | 3 | 5 |
| Benzoic acid, 2-[[3-[[(2,3-dihydro-2-oxo- 1H-benzimidazol-5-yl)amino]carbonyl]-2-hydroxy-1 -naphthalenyl]azo]-, butyl ester | 4 | 0 | 1 | 5 |
| BUMETRIZOLE | 5 | 0 | 0 | 5 |
| 2-(2H-BENZOTRIAZOL-2-YL)-4,6-BIS(1-METHYL-1-PHENYLETHYL)PHENOL | 3 | 0 | 2 | 5 |
| DI-N-PENTYL PHTHALATE (DNPP) | 3 | 0 | 0 | 3 |
| 1,3,5-TRIAZINE-2,4,6(1H,3H,5H)-TRIONE,1,3,5-TRIS((4-(1,1-DIMETHYLETHYL)-3-HYDROXY-2,6-DIMETHYLPHENYL)METHYL)- | 3 | 0 | 0 | 3 |
| 1,3,5-TRIS-[(2S AND 2R)-2,3-EPOXYPROPYL]-1,3,5-TRIAZINE-2,4,6-(1H,3H,5H)-TRIONE | 3 | 0 | 0 | 3 |
| 1-[4-(1,1-dimethylethyl)phenyl]-3-(4-methoxyphenyl)propane-1,3-dione | 3 | 0 | 0 | 3 |
| 6,6'-di-tert-butyl-4,4'-diethyl-2,2'-methylenediphenol | 3 | 0 | 0 | 3 |
| Cadmium selenide | 2 | 0 | 0 | 2 |
| ANOX 20 | 1 | 0 | 0 | 1 |

Table 3: Compiled ecotoxicity data partitioned by primary environmental compartments.
